# Streamflow and water chemistry in cattle‐impacted watersheds of the southern Coastal Plain, USA

**DOI:** 10.1002/jeq2.70239

**Published:** 2026-08-16

**Authors:** Oliva Pisani, Dan Liebert, Earl Keel, Alisa W. Coffin

**Affiliations:** ^1^ USDA‐ARS, Southeast Watershed Research Laboratory Tifton Georgia USA

## Abstract

As part of the long‐term hydrologic monitoring of the Little River Experimental Watershed (LREW) conducted by the United States Department of Agriculture—Agricultural Research Service Southeast Watershed Research Laboratory, precipitation, streamflow, and water chemistry data were collected from four areas of the LREW in southeast Georgia, from 2014 to 2020. Three of the sub‐watersheds are nested and characterized by the presence of cattle. Water chemistry measurements include total suspended solids; dissolved organic carbon and total dissolved nitrogen concentrations; dissolved nutrient species including nitrate + nitrite‐N, ammonia + ammonium‐N, orthophosphate‐P, and chloride; total and dissolved macro‐ and micronutrients including aluminum, arsenic, boron, calcium, cadmium, cobalt, chromium, copper, iron, potassium, magnesium, manganese, molybdenum, sodium, nickel, phosphorus, lead, sulfur, selenium, silicon, titanium, vanadium, and zinc; and dissolved organic matter optical characteristics. Daily streamflow data were paired with biweekly water sample analyte concentrations to calculate monthly analyte loads. Variability in the dataset was observed between the monitoring stations and seasonally within each station. While the dataset has some limitations including gaps in data collection and the lack of proper quality control documentation for 2014, this dataset can be useful to compare trends for various analytes across watersheds of different sizes and with different land cover configurations. The data presented in this article can be used to identify the impacts of land use on streamflow and water chemistry in small cattle‐impacted watersheds.

## Plain Language Summary

Precipitation, streamflow, and water chemistry in four sub‐watersheds of the Little River Experimental Watershed in southeast Georgia were measured from 2014 to 2020. The studied sub‐watersheds are characterized by the presence of cattle. Water chemistry data are described, including total suspended solids, dissolved organic carbon and total dissolved nitrogen concentrations, dissolved nutrient species, total and dissolved macro‐ and micronutrients, and dissolved organic matter characteristics. Daily flow data were paired with biweekly water sample analyte concentrations to calculate monthly analyte loads. This article describes the methods used to collect and analyze water samples. This dataset will be useful to identify the impacts of land cover and land use on streamflow and water chemistry in small cattle‐impacted watersheds.

**Abbreviations**
ARS: Agricultural Research Service
BIX: biological index
CEAP: Conservation Effects Assessment Project
DOC: dissolved organic carbon
DOM: dissolved organic matter
EEM: excitation‐emission matrix
FI: fluorescence index
HIX: humification index
LREW: Little River Experimental Watershed
LTAR: Long‐Term Agroecosystem Research
MDL: method detection limit
SEWRL: Southeast Watershed Research Laboratory
STEWARDS: Sustaining the Earth's Watersheds—Agricultural Research Data System
SUVA: specific UV absorption
TDN: total dissolved nitrogen
TSS: total suspended solids
USDA: United States Department of Agriculture

## 1 INTRODUCTION

Cattle production is a significant component of US agriculture, accounting for over 30% of the total products sold in 2017 (US Department of Agriculture‐National Agricultural Statistics Service [USDA‐NASS], 2019). In the state of Georgia, cattle are a dominant source of income with a total annual market value of $360 million (US Department of Agriculture‐National Agricultural Statistics Service [USDA‐NASS], 2019). Cattle operations can impair water quality in rivers and other water bodies through high loading rates of sediment, nutrients, and pathogens (Hubbard et al., 2004; O'Callaghan et al., 2019). High nutrient concentrations entering streams and lakes may contribute to eutrophication, leading to hypoxia and the formation of harmful algae blooms (Glibert et al., 2018). Animal waste from cattle operations can be a source of microorganisms pathogenic to humans (McAllister & Topp, 2012). When impacted water bodies are used for irrigating crops or recreational activities, elevated concentrations of pathogens pose a potential health hazard. Long‐term hydrologic and water chemistry data can be invaluable for natural resource planning and management of cattle‐impacted watersheds.

The United States Department of Agriculture (USDA)—Agricultural Research Service (ARS) established a regional watershed hydrology research center in Tifton, GA following the publication of a report to the US Senate in 1959 by the Secretary of Agriculture (United States Department of Agriculture [USDA], 1959). The ARS Southeast Watershed Research Laboratory (SEWRL) was established in 1965, and the Little River Experimental Watershed (LREW) was selected as the primary research site. Construction of the LREW hydrologic monitoring network was completed in 1972 with the first streamflow measurements starting in 1967 and water chemistry measurements starting in 1974 (Bosch & Sheridan, 2007). The long‐term data collected from the LREW hydrologic monitoring network have contributed to scientific research evaluating landscape and watershed‐scale hydrologic and water quality processes, precipitation‐runoff relationships, hydrograph characteristics, water yield, and the interactive effects of weather, vegetation, soils, and land use for low‐gradient Coastal Plain streams (Bosch et al., 2021). Related to cattle‐impacted watersheds, research conducted by the SEWRL has focused on quantifying the impacts of dairy cattle liquid waste applications on (1) nitrogen (N) concentrations in surface runoff and groundwater (Hubbard et al., 1987), (2) the denitrification (Lowrance et al., 1995) and nutrient filtering capacity (Vellidis et al., 1993, 1996, 2003) of restored riparian wetlands, and (3) fecal indicator bacteria and pathogens in agricultural ponds (Jenkins et al., 2012, 2015).

In addition to providing data for understanding Coastal Plain watersheds, long‐term datasets from the LREW also contribute to national efforts such as the USDA Natural Resources Conservation Service Conservation Effects Assessment Project (CEAP) and the USDA Long‐Term Agroecosystem Research (LTAR) network. The goal of CEAP is to develop scientific understanding of conservation practice effects at the field and watershed scales to enable regional and national assessments with watershed models (Goodrich et al., 2021; Moriasi et al., 2020). Historical LREW datasets on meteorology (2004–2021) and streamflow (1968–2021) are available through CEAP's web‐based watershed data system STEWARDS (Sustaining the Earth's Watersheds—Agricultural Research Data System, Sadler et al., 2008). As a participant of the LTAR network, the Gulf Atlantic Coastal Plain site contributes data collected in the LREW to enable multidecadal, transdisciplinary, and cross‐location science to ensure the long‐term sustainability of US agriculture (Kleinman et al., 2018). Specifically, LREW datasets have contributed to national scale estimates of water budgets (Baffaut et al., 2020), nutrient budgets (Welikhe et al., 2023), and carbon dioxide fluxes (Menefee et al., 2022) in agroecosystems.

This article describes a unique multidisciplinary dataset (Pisani et al., 2026) collected from 2014 to 2020 in four sub‐watersheds of the LREW in southeast Georgia. Three of the sub‐watersheds are nested (Little River Dairy Wetland [LRDW] within Little River O3 [LRO3] within LRO) and characterized by the presence of cattle. The fourth sub‐watershed is included to serve as a reference watershed (LRN). The dataset includes precipitation, streamflow, and a comprehensive suite of water chemistry variables. Measurements of total suspended solids (TSS), total and dissolved nutrients, heavy metals, and dissolved organic matter (DOM) characteristics provide complementary water quality information about low‐gradient Coastal Plain streams. The data presented in this article will be a valuable resource for researchers and managers identifying the impacts of cattle on water resources.

## 2 SITE DESCRIPTION

**FIGURE 1:**
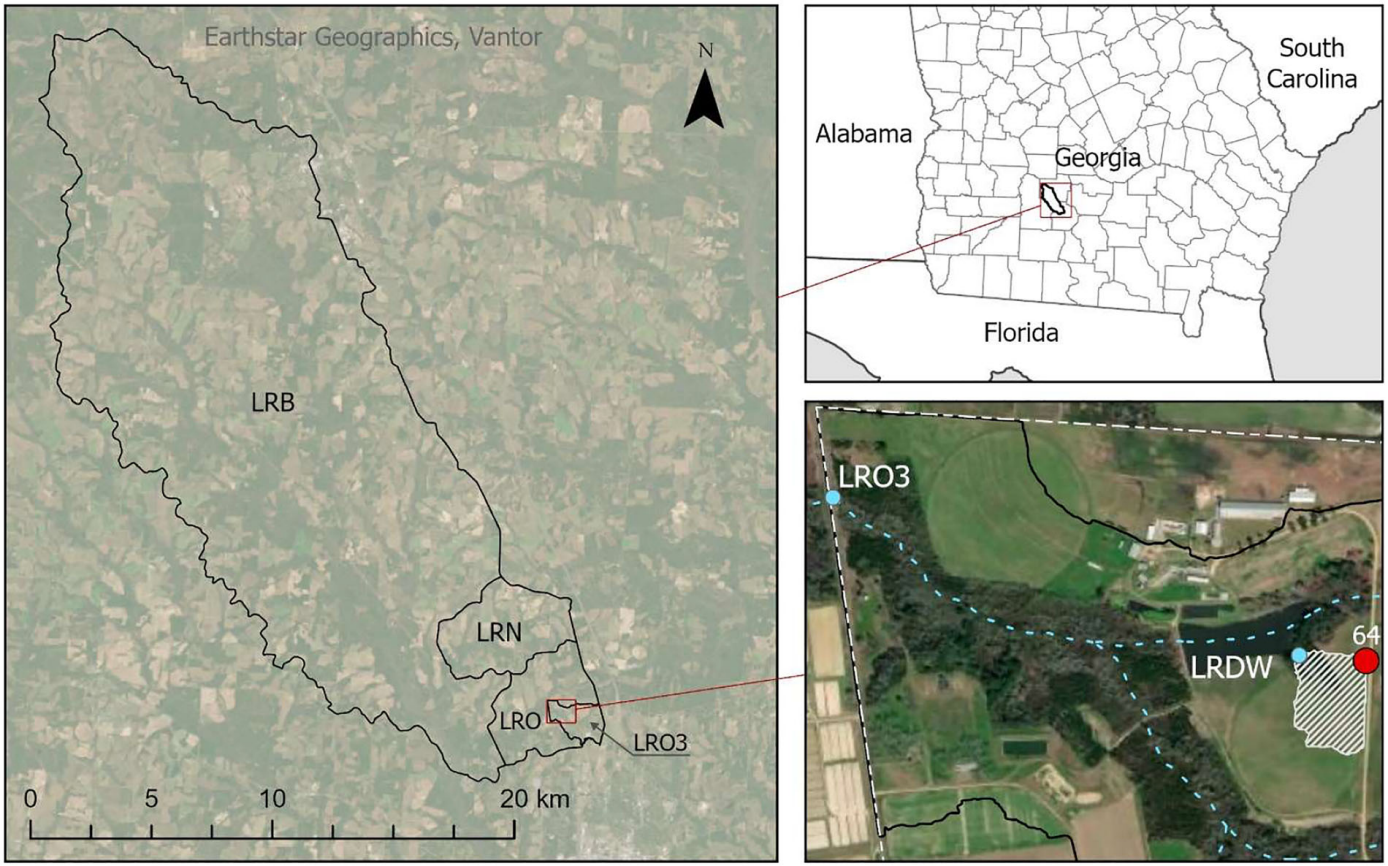
Map of the Little River Experimental Watershed (LREW) showing the entire Little River Basin (LRB) and the four sub‐watersheds described in this study, LRN, LRO, LRO3, and LRDW. The blue circles denote the location of the streamflow measurement stations and autosamplers at each sub‐watershed outlet. The red circle indicates the location of rain gauge #64.

The LREW, located in the Tifton Upland of the Southeastern Plain ecoregion, drains a 33,433‐ha area in the headwaters of the Suwannee River Basin (Figure 1). The hydrology and water quality of the LREW have been monitored by the USDA‐ARS SEWRL since 1967 (Bosch & Sheridan, 2007). Data from four LREW sub‐watersheds are described in this dataset article (Figure 1). The sub‐watersheds Little River N (LRN) and O (LRO) are in the southeast portion of the LREW and are part of the original hydrologic monitoring network. Little River O3 (LRO3) is located at the headwaters of LRO and drains a watershed with a high livestock population from the University of Georgia Animal Science Farm that maintains about 600 cows, calves, and steers. The Little River Dairy Wetland (LRDW) is nested within LRO3 and is on the University of Georgia Animal Science Farm.

The LREW is characterized by broad, flat, alluvial floodplains with low‐gradient, poorly defined channels (Yates, 1976). Upland soils are classified as loamy, siliceous, thermic Plinthic Paleudults, while bottomland soils adjacent to drainage networks are loamy, siliceous, thermic Arenic Plinthic Paleaquults (Calhoun, 1983). The sandy surface soils of the LREW are underlain by the shallow and relatively impermeable Hawthorne formation, which confines the regional Floridian Aquifer. A plinthic layer of dense clay to sandy clay loam with low hydraulic conductivity exists at a depth of 1–2 m, which diverts percolating soil water to the stream network through lateral subsurface flow. In the LREW, surface runoff can vary between 5% and 40% of annual rainfall, while shallow subsurface flow can vary between 2% and 35% (Bosch et al., 2021). Riparian forests that occupy lowland soils in or adjacent to wetlands are an important feature of the LREW that control stream hydrology (Bosch et al., 2014), act as sinks for agrichemical runoff (Hubbard & Lowrance, 1994; Lowrance et al., 1997), and influence stream DOM composition (Pisani et al., 2020). Annual precipitation in the LREW averages 1200 mm, and it is generally greatest in the spring and summer months when convective thunderstorms occur. The mean annual temperature is 19°C, with a monthly mean minimum of 11°C in January and a monthly mean maximum of 27°C in July (Bosch et al., 2017).

Land use across the LREW is a mosaic of cropland (36%), pasture and forage (14%), upland and riparian forest (43%), and wetlands (Bosch et al., 2021). The most common regional crops include cotton, peanuts, and corn. In the LRN sub‐watershed, land use is dominated by cropland (53%), forest and shrubland (35%), and irrigated land (15%), with smaller areas of pasture and herbaceous wetlands (6%; Bosch et al., 2021). Sub‐watershed LRO is like LRN but has a lower percentage of cropland (48%), greater percentage of forest and shrubland (37%), and a lower percentage of irrigated land (9%). The high livestock population within LRO results in a higher percentage of land use under pasture (8%). LRO3 contains a greater fraction of pasture and herbaceous wetlands (10%) and cropland (55%), likely due to the presence of livestock within the watershed (Bosch et al., 2021). The small LRDW sub‐watershed includes a cropped area that is irrigated with liquid dairy waste at a N application rate of 600 kg N/ha/year (Vellidis et al., 2003). Runoff from the cropped area flows through a forested riparian wetland and drains into a constructed holding pond that is approximately 2 ha and holds approximately 2.5 × 10^6^ L (Jenkins et al., 2012).

## 3 DATA COLLECTION AND ANALYSIS

### 3.1 Precipitation

Precipitation data collection in the LREW started in the 1960s and today, the watershed is instrumented to measure precipitation across the 33,433‐ha area using a network of 39 rain gauges (Bosch et al., 2007). Precipitation data for sub‐watersheds LRO3 and LRDW were collected from a monitoring station located within LRO3 (rain gauge 64; 31° 30′ 17.05″ N, 83° 31′ 58.33″ W; Figure 1), which was installed in 1993 and consists of a Texas Electronics TE525 tipping‐bucket rain gauge. The rain gauge has an orifice diameter of 154 mm and can measure 0.254 mm of rainfall per tip. Each tip of the bucket is read by a CR1000 data logger (Campbell Scientific Inc.), converted to rainfall depth, and recorded at 5‐min intervals. Raw data are examined annually and erroneous data removed before summing for daily rainfall totals. Due to the nature of convective thunderstorms, precipitation data for sub‐watersheds LRO and LRN were calculated using area‐weighted means of nearby gauges. The precipitation data record from rain gauge 64 is publicly available on STEWARDS for the period 1993–2021, listed as GALR0064.

### 3.2 Streamflow

The streamflow gauging stations for sub‐watersheds LRN, LRO, and LRO3 were installed at highway box culverts and consist of broad‐crested v‐notch weirs made of rebar‐reinforced concrete poured on a concrete apron. The weirs are located between the outer ends of the upstream culvert wing walls, approximately 3 m upstream from the culvert. The gauging stations consist of 2.5 psi CS451 pressure transducers (Campbell Scientific Inc.), and water levels are recorded with a CR1000 datalogger (Campbell Scientific Inc.) at 5‐min intervals. Extensive geologic and hydrologic assessments were conducted prior to installation of the weirs (Yates, 1976), and these flow measurement structures have previously been described, including the development of rating curves (Bosch & Sheridan, 2007; Bosch et al., 2021). The structural dimensions of the horizontal weir and the depth of the v‐notch vary from station to station (Table 1).

**TABLE 1:** Outlet coordinates (degrees, minutes, seconds [DMS]) and drainage area for each watershed, description of the streamflow measurement device including the weir length and the v‐notch depth, the total period of streamflow and water chemistry record, the total number of water samples collected during the study period (2014–2020), and the percentage of missing samples reported during the study period (2014–2020).

Station	Outlet coordinates (DMS)	Drainage area (ha)	Streamflow measurement device	Weir length (m)	V‐notch depth (cm)	Total period of streamflow record	Total period of water chemistry record	Samples collected (2014–2020)	Missing samples (2014–2020) (%)
LRN	31° 31′ 05″ N, 83° 35′ 11” W	1,567	V‐notch weir	14.8	62.2	Oct. 3, 1970–Jan. 31, 1982; Aug. 1, 2002–Present	Jan. 28, 1974–Dec. 25, 1981; Jan. 7, 2002–Present	135	26
LRO	31° 29′ 37″ N, 83° 34′ 03″ W	1,593	V‐notch weir	14.8	62.5	Nov. 29, 1968–Jan. 31, 1982; Jan. 1, 1993–Present	Jan. 28, 1974–Dec. 30, 1981; Feb. 11, 1993–Present	139	24
LRO3	31° 30′ 27″ N, 83° 32′ 40″ W	275	V‐notch weir	10.7	53.0	Aug. 4, 2007–Present	Apr. 24, 1990–Mar. 10, 1995; Jan. 27, 2004–Present	153	16
LRDW	31° 30′ 17″ N, 83° 32′ 04″ W	2.7	H‐flume	NA	NA	Nov. 25, 2013–Present	Dec. 2, 2013–Present	115	37

At each gauging station, stilling wells are connected hydraulically to the upstream and downstream sides of the structures. Water surface elevation is measured and recorded with a strain‐gauge pressure‐transducer digital datalogger system. The pressure transducers measure the water depth to the nearest 2 mm at 5‐min intervals. The data are stored on dataloggers and transferred to computer storage for processing and review. In the dataset, the 5‐min streamflow values were summed to provide total daily flow.

The LRN weir was removed and rebuilt in 2018 (August 1–September 27) to address ongoing leaks. For the dataset presented in this article, the daily streamflow through LRN during the month when the weir was under construction was estimated using streamflow measured at LRO using the formula:

$$\;\mathrm{Flo}w_{\mathrm{LRN}\;\mathrm{daily}}=\mathrm{Flo}w_{\mathrm{LRO}\;\mathrm{daily}}\;\cdot \frac{\mathrm{Flo}w_{\mathrm{LRN}\;\mathrm{mean}}}{\mathrm{Flo}w_{\mathrm{LRO}\;\mathrm{mean}}}$$

where Flow_LRN mean_ and Flow_LRO mean_ are the average total daily streamflow at stations LRN and LRO for the month prior and the month after the period being estimated, and Flow_LRO daily_ is the daily streamflow measured at LRO. Sub‐watershed LRO was used to estimate daily streamflow at LRN because of the two sub‐watersheds’ adjacency and similar size (Table 1). The original daily streamflow data from LRN and LRO for the total period of record (Table 1) are available for direct download from the STEWARDS database (GALR6550 and GALR6860, respectively). While streamflow from LRO3 has previously been summarized for the period 2007 to 2019 (Bosch et al., 2021), the data have not yet been made available, and the dataset presented here includes streamflow from LRO3 from 2014 to 2020.

At the LRDW sub‐watershed, a 0.45 m (1.5 ft) H‐flume installed at the wetland outlet is used to measure surface water quantity discharged into the farm pond. Tapered earthen berms approximately 10 m long were constructed on either side of the H‐flume to route surface flow leaving the wetland through the flume. Flow depth within the flume is measured with a 2.5 psi CS451 pressure transducer (Campbell Scientific Inc.), and data are recorded with a CR1000 datalogger (Campbell Scientific Inc.) at 5‐min intervals. The University of Georgia operated an H‐flume at this site from 1992 to 2013 (Vellidis et al., 2003), but data from this period are currently unavailable. The dataset presented here is the first report of streamflow data from LRDW since the ARS SEWRL began operating the site in December of 2013 (Table 1).

### 3.3 Sample collection for water chemistry measurements

Water sample collection at the four sub‐watersheds was initiated at different times (Table 1). The methods and frequencies of sample collection have changed over time, reflecting changes in research needs, specific study objectives, laboratory funding levels, and technological advances (Feyereisen et al., 2007). As part of the SEWRL hydrologic monitoring of the LREW, the four sub‐watersheds described in this article are currently sampled once every 2 weeks (biweekly) for water chemistry analyses.

For this study period (2014–2020), water samples were composited biweekly using a 3710 portable sampler (Teledyne ISCO) mounted to a small refrigerator. The refrigerator was maintained at ∼4°C and contained a 9‐L glass sampling container lined with a low‐density polyethylene sampling bag. The sampler pump was set so that a flow‐proportional sample was obtained having the largest number of pumps per sampling period without exceeding the container capacity (9 L). Samples were collected once a minimum composite volume (500 mL) was reached. Due to high intra‐annual variability in flow conditions, this would vary from once every 2 weeks to once in a 4 or 6‐week period during low flow conditions. If a problem occurred with the sampler and the sampling container was empty on the day of collection, a water sample was pumped using the sampler directly into a lined glass sampling container. If a problem occurred with the sampler and the sampling container contained some but not the minimum composite volume (500 mL), additional water sample was pumped using the sampler and combined in the sampling container. If the sampler was malfunctioning, a manual grab sample was collected from the stilling area immediately upstream of the control weir into a 1‐L bottle.

For the study period described in this article (2014–2020), the total number of samples collected was 135, 139, 153, and 115 for LRN, LRO, LRO3, and LRDW, respectively (Table 1). The dataset contains some gaps in sample collection that were due to low flow conditions, technical issues with the samplers, and times when government offices were closed (i.e., the government shutdown, December 21, 2018–February 4, 2019, and the COVID‐19 pandemic, March 3, 2020–April 27, 2020). These instances resulted in 26, 24, 16, and 37% missing samples for LRN, LRO, LRO3, and LRDW, respectively (Table 1).

**FIGURE 2:**
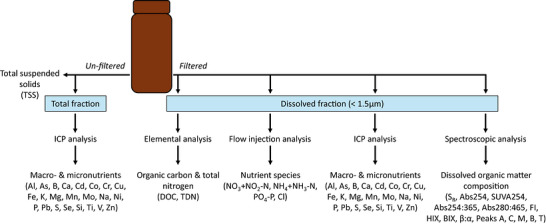
Diagram of water sample processing, analyses performed, and the type of chemistry data generated.

The water chemistry characteristics of each composite sample were assumed to be representative of the mean water chemistry over the biweekly compositing period. For every sampling campaign, a field blank was prepared by placing 2 L of deionized water in a sampling container, transporting it to the field, and treating it as a sample, including exposure to sampling conditions, sample handling, and sample analysis (Pisani et al., 2024). Samples were transported to the lab on ice and immediately split into total and filtered (<1.5 µm) fractions for a suite of chemical analyses: (1) TSS, (2) dissolved organic carbon (DOC) and total dissolved nitrogen (TDN) concentrations, (3) dissolved nutrient species including nitrate + nitrite‐N (NO_3_ + NO_2_‐N), ammonia + ammonium‐N (NH_3_ + NH_4_‐N), orthophosphate‐P (PO_4_‐P), and chloride (Cl), (4) total and dissolved macro‐ and micronutrients including aluminum (Al), arsenic (As), boron (B), calcium (Ca), cadmium (Cd), cobalt (Co), chromium (Cr), copper (Cu), iron (Fe), potassium (K), magnesium (Mg), manganese (Mn), molybdenum (Mo), sodium (Na), nickel (Ni), phosphorus (P), lead (Pb), sulfur (S), selenium (Se), silicon (Si), titanium (Ti), vanadium (V), and zinc (Zn), and (5) DOM optical characteristics (Figure 2). With each biweekly sample set, a laboratory blank (500 mL of deionized water) was prepared, processed, and analyzed the same way as the samples. The number of annual field and laboratory blanks varied from year to year and ranged from a total of 44–54 for the study period (Table 2).

**TABLE 2:** Number of annual field and laboratory blanks; calibration ranges, and annual method detection limits (MDLs) for dissolved organic carbon (DOC), total dissolved nitrogen (TDN), and dissolved nutrient species; total and dissolved macro‐ and micronutrients measured in the water samples, emission wavelengths (Em ʎ) used to quantify them, the percentage detected in the filtered (*n* = 640) and unfiltered (*n* = 579) water samples, the calibration range used for each element, the MDL, and the mean percent recovery of the calibration verification standards. NA, not available.

Blanks		2014	2015	2016	2017	2018	2019	2020
Field		26	24	27	27	27	24	22
Laboratory		26	26	27	27	27	24	22
Total		52	50	54	54	54	48	44

Dissolved organic carbon, total dissolved nitrogen, and dissolved nutrient species								
		Method detection limit (mg/L)						
Analyte	Calibration range (mg/L)	2014	2015	2016	2017	2018	2019	2020
DOC	0–50	NA	NA	0.558	0.285	0.867	1.17	0.759
TDN	0–10	0.0719	NA	0.0640	0.0502	0.130	0.158	0.111
NO_3_ + NO_2_‐N	0.005–2	0.0599	0.0722	0.0281	0.008	0.00645	0.00608	0.00683
NH_3_ + NH_4_‐N	0.005–2.5	0.0357	0.0185	0.0197	0.0145	0.029	0.00677	0.00814
PO_4_‐P	0.005–2	0.0307	0.0113	0.00717	0.00623	0.00668	0.005	0.00761
Cl	0.05–20	0.674	0.309	0.328	0.146	0.16	0.0699	0.194

Total and dissolved macro‐ and micronutrients						
Analyte	Em ʎ (nm)	Filtered % detected	Unfiltered % detected	Calibration range (mg/L)	Method detection limit (mg/L)	Recovery (%)
Al	308.2	83	97	0.1–100	0.1	100
As	193.7	3	3	0.01–10	0.01	101
B	249.7	10	8	0.01–10	0.0356	95
Ca	317.9	100	100	0.01–100	0.336	98
Cd	226.5	0	1	0.001–10	0.001	102
Co	230.7	9	43	0.001–10	0.001	97
Cr	205.5	0	10	0.001–10	0.00763	101
Cu	224.7	9	16	0.01–100	0.01	101
Fe	238.2	100	100	0.1–100	0.1	97
K	766.4	100	100	0.1–100	0.197	100
Mg	279.0	100	100	0.01–100	0.0457	102
Mn	257.6	77	99	0.001–100	0.00233	97
Mo	202.0	2	2	0.001–10	0.00558	103
Na	589.5	100	100	0.1–100	0.321	99
Ni	231.6	0	3	0.001–10	0.00682	103
P	178.2	100	100	0.01–100	0.01	105
Pb	220.3	19	53	0.001–10	0.00152	102
S	182.0	100	100	0.01–100	0.244	103
Se	196.0	0	0	0.01–10	0.01	102
Si	251.6	98	98	0.1–100	1.57	97
Ti	334.9	93	94	0.001–10	0.00229	98
V	292.4	47	68	0.001–10	0.00167	94
Zn	213.8	3	16	0.001–10	0.0389	101

### 3.4 Total suspended solids

Prior to sample filtration, glass fiber filters (Whatman 934‐AH, 1.5 µm) were rinsed with 100 mL of deionized water, dried at 105°C for at least 24 h, and stored in a desiccator until used. TSS concentration was measured by passing a 600 mL aliquot of each sample through a pre‐weighed glass fiber filter and measuring the oven dry weight (dried at 105°C overnight) of the material trapped on the filter. The TSS concentration, including organic and inorganic constituents, was calculated by dividing the mass of the material by the filtered sample volume (Dalzell & Pisani, 2024). The field and laboratory blanks often resulted in negative TSS values, likely due to the removal of additional material from the filters during filtration. For this reason, a blank‐based method detection limit (MDL) could not be calculated, and the TSS detection limit was based on the precision of the analytical balance used (capable of reading to 0.00001 g or 0.01 mg). In the dataset described in this article, TSS values ranged from 0.333 mg/L at LRDW to 3330 mg/L at LRO3. While no TSS values fell below the practical quantitation limit, users of the dataset should be aware that reproducibility may be poor for samples with low TSS. The filtered water sample was split into subsamples for subsequent analyses (Figure 2).

### 3.5 Dissolved organic carbon and total dissolved nitrogen

Conventionally, a 0.45‐µm pore size filter is used to distinguish between particulate and dissolved C and N fractions in water. Because a 1.5‐µm filter was used in this study, the reported DOC and TDN values include colloidal (0.45–1.2 µm) and fine particulate (1.2–1.5 µm) materials. The filter pore size must be taken into consideration when comparing this dataset to other laboratory and field studies. From 2016 to 2020, a filtered subsample was analyzed for DOC (Figure 2) by high‐temperature catalytic oxidation (Standard Method 5310B) on a Shimadzu TOC‐VCPN analyzer with an ASI‐V autosampler (Shimadzu Scientific Instruments). The instrument was calibrated from 0 to 50 mg C/L using a potassium hydrogen phthalate standard. The 50 mg C/L standard was run in the middle of the batch to confirm calibration stability. Filtered subsamples were analyzed for TDN in 2014 and from 2016 to 2020. In 2014, concentrations were measured on a Lachat QuikChem 8500 flow injection analyzer (QuikChem AE, Lachat Chemicals Inc.) using in‐line persulfate digestion (QuikChem Method 31‐107‐04‐4‐A). Samples were analyzed in duplicate. If duplicates did not agree within 10% of each other, they were reanalyzed. From 2016 to 2020, concentrations were measured using a Shimadzu TOC‐VCPN analyzer with an ASI‐V autosampler and a TNM‐1 N module (Shimadzu Scientific Instruments). The instrument was calibrated from 0 to 10 mg N/L using a potassium nitrate standard. The 10 mg N/L standard was run in the middle of the batch to confirm calibration stability. The annual DOC and TDN concentrations from the field and laboratory blanks were used to calculate MDLs by summing the annual mean and the standard deviation of the blank results and multiplying by the Student's *t*‐value (EPA 821‐R‐16‐006, Table 2). Measurements of DOC and TDN that were zero or greater but below the annual MDL were included in the dataset, allowing users to decide how to process these non‐detect values (e.g., replace them with zeros or a fraction of the MDL). Negative values were replaced with zero.

### 3.6 Dissolved nutrient species

Another filtered subsample was analyzed for nutrient species including NO_3_ + NO_2_‐N, NH_3_ + NH_4_‐N, PO_4_‐P, and Cl (Figure 2) using US EPA approved colorimetric techniques (EPA 353.2, 350.1, 365.1, and 9251, respectively) on a Lachat QuikChem 8500 flow injection analyzer (QuikChem AE, Lachat Chemicals Inc.). The filter pore size used in this study (1.5 µm) can impact nutrient stability, particularly for PO_4_‐P, by including microparticulate surfaces on which adsorption–desorption reactions can occur (Haygarth et al., 1995). The filter pore size must be taken into consideration when comparing this dataset to other laboratory and field studies. All samples were run in duplicate and duplicate analyses that exceeded a 10% difference were reanalyzed. Beginning in 2015, the instrument was calibrated (Hach Standards) using 8–10 calibration points at appropriate ranges for each analyte (Table 2). Mid‐range check standards included a wastewater effluent quality control standard for NO_3_‐N, NH_4_‐N, and PO_4_‐P, and a Cl standard prepared by dissolving a known amount of potassium chloride (KCl) in deionized water. Check standards were run after every 10–12 unknowns to confirm the analyses were accurate within 10% of the target concentration. Samples with analyte responses greater than the highest calibration point were diluted within the calibration range and reanalyzed. The annual nutrient concentrations from the field and laboratory blanks were used to calculate MDLs as described above (EPA 821‐R‐16‐006, Table 2). Changes in calibration methods for flow injection analyzers applied in 2015 resulted in lower detection limits and increases in reportable nonzero concentrations compared to historical datasets (1974–2014). If the concentration of dissolved nutrient species was zero or greater but below the annual MDL, the measured values were included in the dataset. Negative values were replaced with zero.

### 3.7 Total and dissolved macro‐ and micronutrients

Filtered and unfiltered subsamples were analyzed for a suite of macro‐ and micronutrients (Al, As, B, Ca, Cd, Co, Cr, Cu, Fe, K, Mg, Mn, Mo, Na, Ni, P, Pb, S, Se, Si, Ti, V, and Zn, Figure 2) using Inductively Coupled Plasma with Optical Emission Spectroscopy (ICP‐OES). Due to the filter pore size used for the filtered subsamples (1.5 µm), the dissolved phase also includes colloids and microparticles, which can cause analyte losses through adsorption of trace elements and heavy metals on surfaces (Mester & Sturgeon, 2003). Unfiltered water samples were available from 2015 to 2020, so total macro‐ and micronutrient concentration data are not available for 2014. Samples (40 g) were reduced in volume and digested (EPA 200.2, modified). Modifications to the digestion method include adding 1 mL of concentrated trace metal grade nitric acid (HNO_3_) to the samples and reducing the sample volume on a HotBlock digestion system (Environmental Express) set to 115°C. Once the sample volume was reduced to ∼12 mL, 3 mL of concentrated trace metal grade hydrochloric acid (HCl) was added to the samples, which were refluxed by heating at 125°C for 1 h on the same HotBlock. For a more thorough digestion, 1 mL of hydrogen peroxide (H_2_O_2_) was added to each sample and refluxed for an additional 30 min. Two reagent blanks and two known control samples were digested with each batch of approximately 40 samples. Sample digestates were quantitatively transferred to centrifuge tubes and diluted to 15 mL with 2% HNO_3_ (prepared with laboratory grade deionized water). The digestates were analyzed using an iCAP 7400 ICP‐OES Duo equipped with a Charge Injection Device detector (Thermo Fisher Scientific). An aliquot of digested sample was aspirated from the centrifuge tube using a CETAC ASX‐520 autosampler (Teledyne CETAC Technologies) and passed through a concentric tube nebulizer. The resulting aerosol was then swept through the plasma using argon as the carrier gas with a flow rate of 0.5 L/min and a nebulizer gas flow rate of 0.7 L/min. Macro‐ and micronutrients were quantified by monitoring the emission wavelengths reported in Table 2.

The standards used for instrument calibration were prepared gravimetrically and nutrient concentrations were determined in mg/L. A calibration curve was run at the beginning of each sequence with calibration ranges from 0.001 to 100 mg/L. Because environmental concentrations and instrumental sensitivity vary among elements, the different macro‐ and micronutrients required varying calibration ranges (Table 2). Calibration verification standards were run every 12 (1 mg/L) and 24 (10 mg/L) samples to monitor the stability and accuracy of the calibration. Calibration and calibration verification standards were diluted with reagent blanks that were digested in the HotBlock using the sample digestion protocol. The mean percent recovery of the calibration verification standards ranged from 94% for V to 105% for P (Table 2). All measurements of standards and samples were performed in triplicate. A blank‐based MDL was calculated using all the filtered and unfiltered field and laboratory blanks digested and analyzed for this study (*n* = 336). The MDL for each analyte was determined as the greater of the blank‐based MDL and the low calibration point for that analyte (Table 2). If the nutrient concentration was zero or greater but below the MDL, the measured values were included in the dataset. Negative values were replaced with zero.

### 3.8 Dissolved organic matter

Filtered subsamples used for DOM optical characterizations (Figure 2) were stored in pre‐washed 60‐mL brown Nalgene bottles kept in a refrigerator (4°C) until analysis by UV‐visible and fluorescence spectroscopy. DOM is operationally defined as organic matter passing a 0.45‐µm pore size filter (Nebbioso & Piccolo, 2013). When this work began, the filter pore size was 1.5 µm, resulting in samples that included dissolved (<0.45 µm), colloidal (0.45–1.2 µm), and fine particulate (1.2–1.5 µm) organic matter fractions. Suspended particles can absorb and scatter light, thus influencing spectroscopic measurements of DOM optical properties (Massicotte et al., 2017). For this reason, the filter pore size must be taken into consideration when comparing DOM optical data among studies or laboratories.

Samples were removed from refrigeration and warmed to room temperature just before analysis. Chemical characteristics of the DOM were assessed through the analysis of optical properties on an Aqualog spectrofluorometer (Horiba Scientific) equipped with a 150 W continuous output xenon arc lamp. Excitation‐emission matrix (EEM) scans were acquired in a 1 cm quartz cuvette with excitation wavelengths (Ex ʎ) scanned using a double‐grating monochrometer from 240 to 621 nm at 3‐nm intervals. Emission wavelengths (Em ʎ) were scanned from 246 to 693 at 2‐nm intervals, and emission spectra were collected using a charge‐coupled device detector. All fluorescence spectra were acquired in sample‐over‐reference ratio mode to account for potential fluctuations and wavelength dependency of the lamp output. Each sample EEM underwent spectral subtraction of a deionized water blank to remove the effects due to Raman scattering. Samples were corrected for the inner filter effect and Rayleigh masking was applied to remove the signal intensities for both the first and second order Rayleigh lines. The fluorescence intensities were normalized to the area under the water Raman peak collected on each day of analysis and are expressed in Raman‐normalized intensity units. All sample EEM processing was performed with the Aqualog software (version 4.0.0.86).

**FIGURE 3:**
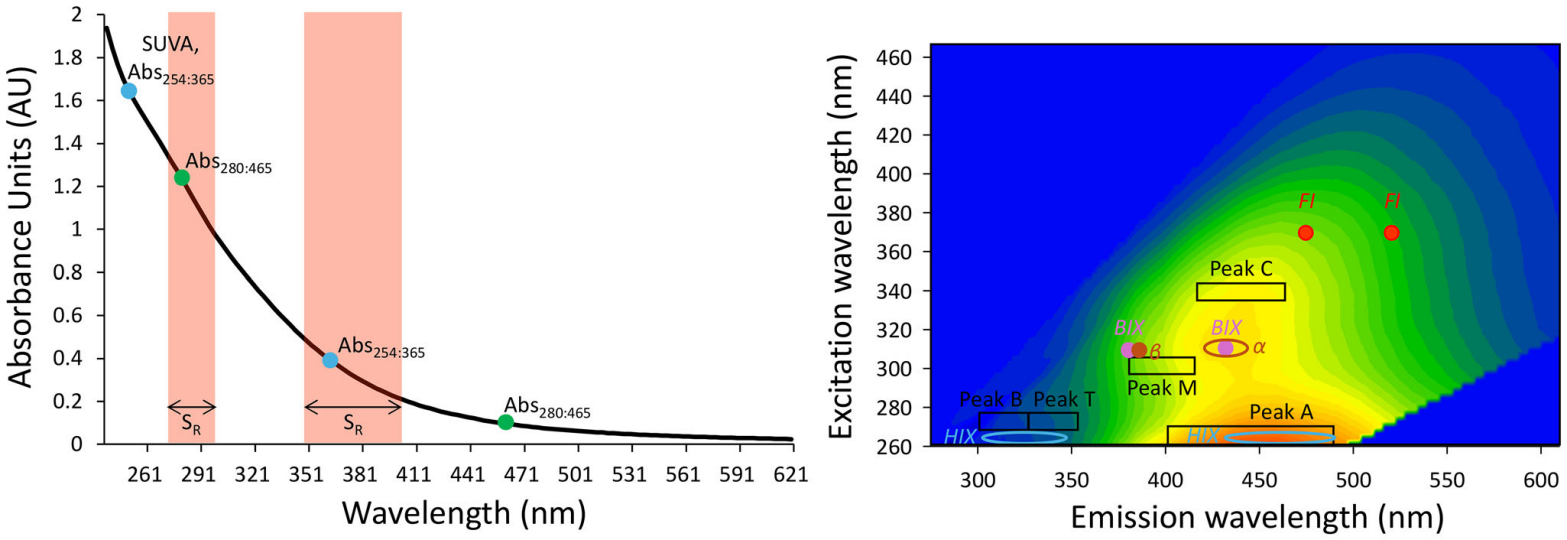
Optical indices derived from absorbance and excitation‐emission matrix (EEM) fluorescence scans used to analyze the composition of dissolved organic matter (DOM).

The optical data obtained from the absorbance and EEM scans were used to calculate several indices indicative of DOM chemical composition (Hansen et al., 2018 and references therein, Figure 3). From the absorbance data, the slope ratio (*S_R_*), absorbance at 254 nm (Abs254), the specific UV absorbance at 254 nm (SUVA254), the ratio of the absorbance at 254 and 365 nm (Abs254:365), and the ratio of the absorbance at 280 and 465 nm (Abs280:465) were calculated (Figure 3). The *S_R_* was calculated as the ratio of the 275–295 nm and the 350–400 nm spectral slope regions of the absorbance spectra. The SUVA254 was calculated by dividing the absorbance at 254 nm by the DOC concentration of the sample. From the EEM data, the fluorescence index (FI), humification index (HIX), biological index (BIX), and the freshness index (β:α) were calculated (Figure 3). The FI was calculated as the ratio of the emission intensities at 470 and 520 nm at an Ex ʎ of 370 nm. The HIX was obtained by dividing the area under the emission spectrum from 435 to 480 nm by the sum of the areas under the emission spectrum from 300 to 345 nm and from 435 to 480 nm, at an Ex ʎ of 254 nm. The BIX was calculated as the ratio of emission intensities at 380 and 430 nm, at an Ex ʎ of 310 nm. The freshness index β:α was calculated by diving the emission intensity at 380 nm by the maximum emission intensity between 420 and 435 nm, at an Ex ʎ of 310 nm. To further characterize the DOM, the fluorescence intensity at specific excitation‐emission wavelength pairs was quantified (Figure 3): Peak A (Ex ʎ 260 and Em ʎ 450), Peak C (Ex ʎ 340 and Em ʎ 440), Peak M (Ex ʎ 300 and Em ʎ 390), Peak B (Ex ʎ 275 and Em ʎ 310), and Peak T (Ex ʎ 275 and Em ʎ 340).

### 3.9 Analyte load calculations

Streamflow analyte loads were calculated as follows:

$$\mathrm{Analyte}\;\mathrm{load}\;=\left[\mathrm{Analyte}\;\mathrm{concentration}\right]\;\times \left[\mathrm{streamflow}\;V\right]$$

The analyte concentration (mg/L) and the streamflow volume (L) were multiplied for each daily value. To extend the usefulness of the data, analyte loads were calculated as daily values regardless of the sampling interval. This approach requires certain assumptions about stream analyte concentrations on non‐sample dates, analyte concentrations from manually collected samples, and during periods of missing data. If there were sequential flow composite samples, the analyte concentration of the composites was applied to all the days during that sampling period except the sampling day. For the sampling day, the concentration used was the average of the samples that ended and began on that day. Samples collected manually (using the sampler pump and manual grab samples) were treated the same as flow composites. When an insufficient sample volume was found in the sampling container on the day of sample collection and additional sample was pumped and combined, the concentration measured in the sample was used for the sampling day, and the average of the pumped sample and the previous sample was applied to the sampling period preceding the sampling day.

The number of missing samples over the 7‐year study period ranged from 16% at LRO3 to 37% at LRDW (Table 1). To calculate loads, the missing concentrations were filled as follows: If streamflow was measured but no sample was collected, the average concentration of the preceding and the succeeding sampling periods was used. If the missing sample occurred at the end of the study period (2020), the analyte concentration from the previous sample was used. This sometimes resulted in using the same concentration to fill gaps for several months, as in the case of LRO where only two samples were collected from June 23 to December 31, 2020. At the LRDW station, one sample was collected approximately every 2 months from June 13 to December 6, 2016, and the concentration of each sample was used to fill in the missing data for the preceding sampling period. During periods of government closures (i.e., the government shutdown and COVID‐19 pandemic), the samples were left refrigerated in the field, and the analyte concentration of those samples was used. Concentrations measured below the MDL for a particular analyte were included in the dataset to minimize the need for substituting data points with zeros or a fraction of the MDL, which can obscure patterns and trends in environmental chemistry datasets (Helsel, 2006). Concentrations measured above zero but below the analyte MDL were included and used for load calculations.

Monthly analyte loads (kg/ha/month) were calculated by summing the products of the daily analyte concentrations and daily streamflow volumes over 1‐month periods and dividing by the area of the watershed (ha) to normalize for watershed area. Previous studies conducted in the LREW have summed loads over seasons (Feyereisen et al., 2008) or years (Bosch et al., 2020; Lowrance & Leonard, 1988; Lowrance et al., 1985). Because zero flow volumes result in zero analyte loads, loads were calculated as monthly totals to reduce datapoints equaling zero while increasing temporal resolution. The improved temporal resolution should improve the utility of the dataset for elucidating system dynamics.

## 4 DATASET VARIABILITY

**FIGURE 4:**
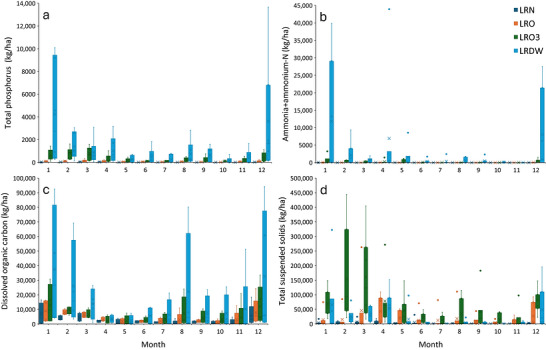
Boxplots comparing the monthly loads (kg/ha) averaged over the study period (2014–2020) for total phosphorus (a), ammonia + ammonium − nitrogen (b), dissolved organic carbon (c), and total suspended solids (d). The line within each box indicates the median. The × indicates the mean. The whiskers extend from the box to the minimum and maximum values. Outliers are plotted as individual points beyond the whiskers.

Table 3 summarizes the observations of precipitation, streamflow, TSS, and all the chemical analytes measured in the filtered and unfiltered samples collected from the four LREW sub‐watersheds from 2014 to 2020. The average and standard deviation values were calculated, including the analyte concentrations above zero but below the MDL. This dataset allows the comparison of a small watershed that receives surface and subsurface runoff primarily from a field irrigated with dairy waste (LRDW), a station downstream of a 2‐ha impoundment and a heavily vegetated 1.02‐km stream reach (LRO3), a station 3.51‐km further downstream (LRO), and an adjacent watershed that is comparable in size to LRO (LRN). The variability in analyte concentrations reported within each station (Table 3) may be a result of the interaction between geochemical factors such as natural buffering capacity and pH that can impact nutrient solubility, hydrological factors that modulate nutrient transport, dilution and resuspension, and management practices (Biedunkova et al., 2025) such as the dairy liquid waste applications at LRDW. Most of the variability in the dataset is between the flow‐monitoring stations, illustrated by monthly comparisons of total P and NH_3_ + NH_4_‐N loads at the four stations (Figure 4a,b). As illustrated by monthly comparisons of DOC and TSS loads at the four stations (Figure 4c,d), a secondary aspect of the variability is temporal, that is, month‐to‐month or seasonal variability within each station that may reflect seasonal changes in rainfall and evapotranspiration rates of the riparian forests that control streamflow and nutrient loading of the watersheds. This dataset article provides a detailed description and summary of the precipitation, streamflow, and water chemistry data reported by Pisani et al., 2026. While not within the scope of this article, the application of advanced statistical techniques can be instrumental in unraveling sources and controlling factors of nutrient distributions in these four small sub‐watersheds of the LREW.

**TABLE 3:** Study period (2014–2020) averages ± standard deviations for the analytes measured at the four Little River Experimental Watershed (LREW) sub‐watersheds LRN, LRO, Little River O3 (LRO3), and Little River Dairy Wetland (LRDW). These statistics include the analyte concentrations above zero but below the method detection limit (MDL). For dissolved and total macro‐ and micronutrients, only the elements detected in >50% of the samples are summarized.

	Units	LRN	LRO	LRO3	LRDW
Precipitation	mm/year	1249 ± 201	1235 ± 214	1209 ± 251	1209 ± 251
Streamflow	mm/year	303 ± 136	336 ± 96.5	493 ± 272	500 ± 258
TSS	mg/L	8.94 ± 14.8	85.7 ± 223	282 ± 520	52.9 ± 93.5
Total dissolved nutrients					
DOC	mg/L	15.9 ± 6.80	16.3 ± 5.13	20.8 ± 8.05	43.5 ± 22.6
TDN	mg/L	1.21 ± 5.26	1.02 ± 0.494	3.10 ± 2.47	11.8 ± 18.0
Dissolved nutrient species					
NO_3_ + NO_2_‐N	mg/L	0.184 ± 0.214	0.413 ± 0.464	1.76 ± 1.47	5.62 ± 8.00
NH_3_ + NH_4_‐N	mg/L	0.050 ± 0.132	0.079 ± 0.137	0.504 ± 0.997	8.19 ± 23.9
PO_4_‐P	mg/L	0.012 ± 0.035	0.038 ± 0.067	0.253 ± 0.867	2.17 ± 2.54
Cl	mg/L	11.9 ± 2.46	14.1 ± 3.82	20.2 ± 7.22	42.1 ± 27.3
Dissolved macro‐ and micronutrients					
Al	mg/L	0.960 ± 1.46	0.518 ± 0.555	0.557 ± 0.732	0.596 ± 0.609
Ca	mg/L	7.72 ± 1.55	11.1 ± 4.35	15.9 ± 5.11	19.4 ± 16.9
Fe	mg/L	1.28 ± 0.643	0.947 ± 0.595	1.07 ± 0.736	0.435 ± 0.200
K	mg/L	3.91 ± 0.604	5.81 ± 1.68	10.8 ± 3.91	40.7 ± 48.7
Mg	mg/L	3.99 ± 0.808	4.58 ± 1.02	7.78 ± 1.70	15.0 ± 11.2
Mn	mg/L	0.027 ± 0.085	0.016 ± 0.066	0.020 ± 0.078	0.018 ± 0.044
Na	mg/L	4.46 ± 0.882	5.36 ± 1.45	10.9 ± 3.86	35.1 ± 21.0
P	mg/L	0.036 ± 0.019	0.084 ± 0.039	0.265 ± 0.169	2.66 ± 2.97
S	mg/L	1.22 ± 0.783	1.46 ± 0.698	1.79 ± 1.24	3.97 ± 6.69
Si	mg/L	3.79 ± 1.22	3.79 ± 0.903	3.88 ± 1.21	3.90 ± 1.60
Ti	mg/L	0.023 ± 0.030	0.014 ± 0.014	0.015 ± 0.018	0.012 ± 0.013
Dissolved organic matter					
*S_R_*		0.819 ± 0.0826	0.793 ± 0.0485	0.773 ± 0.0676	0.759 ± 0.0576
Abs254		0.525 ± 0.115	0.527 ± 0.144	0.665 ± 0.239	1.49 ± 0.477
SUVA254	L/mg C/m	3.63 ± 1.40	3.47 ± 1.34	3.53 ± 1.79	3.97 ± 2.14
Abs254:365		4.40 ± 0.549	4.53 ± 0.354	4.45 ± 0.373	4.22 ± 0.363
Abs280:465		15.3 ± 3.51	16.1 ± 2.75	15.7 ± 2.60	14.5 ± 2.77
FI		1.49 ± 0.0286	1.49 ± 0.0352	1.50 ± 0.0804	1.46 ± 0.167
HIX		0.909 ± 0.0244	0.906 ± 0.0143	0.906 ± 0.0220	0.917 ± 0.0665
BIX		0.582 ± 0.0262	0.586 ± 0.0318	0.608 ± 0.0349	0.605 ± 0.0872
β:α		0.575 ± 0.0260	0.579 ± 0.0319	0.603 ± 0.0356	0.596 ± 0.0828
Peak A	RU	14.3 ± 10.4	13.3 ± 11.0	15.9 ± 14.3	36.3 ± 28.9
Peak C	RU	8.66 ± 6.31	8.28 ± 6.92	9.95 ± 9.16	23.7 ± 19.8
Peak M	RU	6.17 ± 4.51	5.85 ± 4.82	7.15 ± 6.38	16.1 ± 13.0
Peak B	RU	0.876 ± 0.951	0.815 ± 0.834	1.31 ± 2.31	0.948 ± 2.50
Peak T	RU	2.16 ± 1.67	2.15 ± 1.80	3.03 ± 3.52	6.09 ± 5.69
Total macro‐ and micronutrients					
Al	mg/L	1.29 ± 2.10	3.16 ± 6.38	9.61 ± 14.2	1.91 ± 2.79
Ca	mg/L	7.82 ± 1.61	12.0 ± 5.98	17.9 ± 6.78	21.2 ± 18.6
Fe	mg/L	1.90 ± 1.16	3.32 ± 5.13	11.9 ± 19.5	1.04 ± 1.16
K	mg/L	3.94 ± 0.650	5.95 ± 1.78	10.9 ± 4.10	43.4 ± 44.7
Mg	mg/L	4.07 ± 0.815	4.81 ± 1.14	8.31 ± 2.14	16.4 ± 12.4
Mn	mg/L	0.107 ± 0.121	0.144 ± 0.283	0.330 ± 0.423	0.049 ± 0.072
Na	mg/L	4.44 ± 0.849	5.41 ± 1.46	10.6 ± 3.75	36.5 ± 20.6
P	mg/L	0.061 ± 0.041	0.254 ± 0.325	1.35 ± 1.76	3.59 ± 4.34
S	mg/L	1.21 ± 0.813	1.50 ± 0.685	1.91 ± 0.913	4.39 ± 6.89
Si	mg/L	3.94 ± 1.55	5.70 ± 4.73	8.25 ± 4.41	4.91 ± 2.39
Ti	mg/L	0.014 ± 0.020	0.033 ± 0.058	0.093 ± 0.117	0.021 ± 0.030

## 5 SUMMARY

This article reports precipitation, streamflow, and water chemistry datasets collected from 2014 to 2020 in four sub‐watersheds (LRN, LRO, LRO3, and LRDW) of the LREW in southeast Georgia (Pisani et al., 2026). This dataset has four main limitations, including (1) some significant gaps in sample collection, (2) lack of proper quality control documentation for chemistry data collected in 2014, (3) not recording the true end dates for the biweekly samples, and (4) not using an appropriate filter for the analysis of the true dissolved fraction (<0.45 µm). (1) The sample collection gaps typically occurred due to low flow conditions, malfunction of the sampler, or periods of time when the government offices were closed. As a result, missing concentration values had to be filled in, often resulting in the same concentration value being used for multiple months. This gap‐filling method can add uncertainty by smoothing out concentration data and missing extreme values such as high load spikes or rapid fluctuations, typically resulting in an underestimation of total nutrient loads (van't Veen et al., 2025). More sophisticated filling methods such as weighted regressions on time, discharge, and season could not be applied to this dataset due to the small sample size (required to be >200 samples) and the short period of sample collection (required to be >20 years; Hirsch et al., 2010). As a result of these sample collection gaps, caution must be taken when estimating analyte loads from these watersheds. (2) The lack of documentation of quality assurance and quality control methods impacts samples analyzed prior to 2015. Since then, there has been consistent application and documentation of quality control methods. (3) Although the sampling period is once every 2 weeks, the sampler sometimes filled the sampling container before the collection date. However, the true sampling end date was never recorded, and the collection date was used as the end date. Beginning in June 2020, the true end date has been recorded, providing a more precise sampling period to apply to sample concentrations. (4) Because a 1.5‐µm pore size filter was used during this study period, the water samples contained dissolved, colloidal, and fine particulate material. Suspended colloids and microparticles in the samples can impact nutrient stability by providing surfaces on which adsorption–desorption reactions can occur and can attenuate light and impact spectroscopic measurements for DOM characterization. Users of the water chemistry data should use caution when comparing values reported in this dataset with field or laboratory studies using different pore size filters.

Although this dataset has some limitations, such long‐term datasets have value for researchers and are essential for determining the impact of agricultural management on regional water resources. This and the historical LREW precipitation, streamflow, and water chemistry data have been used in the development of water yield and water balance information (Bosch et al., 2021), hydrologic and water quality budgets (Bosch et al., 2017, 2020; Feyereisen et al., 2008; Lowrance et al., 1985), rainfall‐streamflow relationships (Bosch et al., 2006), understanding the impacts of riparian buffers on water quality (Bosch et al., 2014; Hubbard & Lowrance, 1994; Lowrance et al., 1995; Pisani et al., 2020; Vellidis et al., 2002, 2003), and to support natural resource and environmental quality model testing and simulations in the Coastal Plain (Cho et al., 2010). The dataset presented in this article includes 6 years of complementary water chemistry measurements and can broadly be used to compare trends for various analytes across watersheds of different sizes and with different land cover configurations. More specifically, the dataset can be valuable for quantifying nutrient load attenuation in nested watersheds starting from a site receiving liquid dairy waste applications. The DOM portion of the dataset can be used to distinguish between natural (e.g., soil and plant) and anthropogenic DOM sources (e.g., agricultural runoff), to assess eutrophication by quantifying microbial‐ and algae‐derived DOM components, and to estimate carbon flow and cycling in the LREW. The dataset highlights the importance of monitoring DOM optical indices for an evaluation of DOM biogeochemistry (sources, composition, and reactivity) in agricultural watersheds.

## AUTHOR CONTRIBUTIONS

**Oliva Pisani**: Conceptualization; data curation; formal analysis; investigation; methodology; project administration; supervision; visualization; writing—original draft. **Dan Liebert**: Data curation; formal analysis; investigation; methodology; validation; visualization; writing—original draft. **Earl Keel**: Data curation; methodology; validation; writing—review and editing. **Alisa W. Coffin**: Funding acquisition; resources; supervision; writing—review and editing.

## CONFLICT OF INTEREST STATEMENT

The authors declare no conflicts of interest.

## Data Availability

The precipitation, streamflow, and water chemistry and monthly analyte load datasets described in this article are available as comma‐separated (csv) files through the USDA Ag Data Commons (Pisani et al., 2026). The precipitation data from rain gauge 64 are also available on the USDA CEAP STEWARDS database. Streamflow data for LRN and LRO are available on the STEWARDS database along with more detailed metadata.
